# Widespread roles for piRNAs and WAGO-class siRNAs in shaping the germline transcriptome of *Caenorhabditis elegans*

**DOI:** 10.1093/nar/gkz1178

**Published:** 2019-12-24

**Authors:** Kailee J Reed, Joshua M Svendsen, Kristen C Brown, Brooke E Montgomery, Taylor N Marks, Tarah Vijayasarathy, Dylan M Parker, Erin Osborne Nishimura, Dustin L Updike, Taiowa A Montgomery

**Affiliations:** 1 Department of Biology, Colorado State University, Fort Collins, CO 80523, USA; 2 Cell and Molecular Biology Program, Colorado State University, Fort Collins, CO 80523, USA; 3 Department of Biochemistry and Molecular Biology, Colorado State University, Fort Collins, CO 80523, USA; 4 Mount Desert Island Biological Laboratory, Bar Harbor, ME 04672, USA

## Abstract

Piwi-interacting RNAs (piRNAs) and small interfering RNAs (siRNAs) are distinct classes of small RNAs required for proper germline development. To identify the roles of piRNAs and siRNAs in regulating gene expression in *Caenorhabditis elegans*, we subjected small RNAs and mRNAs from the gonads of piRNA and siRNA defective mutants to high-throughput sequencing. We show that piRNAs and an abundant class of siRNAs known as WAGO-class 22G-RNAs are required for proper expression of spermatogenic and oogenic genes. WAGO-class 22G-RNAs are also broadly required for transposon silencing, whereas piRNAs are largely dispensable. piRNAs, however, have a critical role in controlling histone gene expression. In the absence of piRNAs, histone mRNAs are misrouted into the nuclear RNAi pathway involving the Argonaute HRDE-1, concurrent with a reduction in the expression of many histone mRNAs. We also show that high-level gene expression in the germline is correlated with high level 22G-RNA production. However, most highly expressed genes produce 22G-RNAs through a distinct pathway that presumably involves the Argonaute CSR-1. In contrast, genes targeted by the WAGO branch of the 22G-RNA pathway are typically poorly expressed and respond unpredictably to loss of 22G-RNAs. Our results point to broad roles for piRNAs and siRNAs in controlling gene expression in the *C. elegans* germline.

## INTRODUCTION

Piwi-interacting RNAs (piRNAs) and small interfering RNAs (siRNAs) are discrete classes of small RNAs with largely non-overlapping genetic requirements, but which share certain biological functions, such as transposon silencing (1–3). The extent to which piRNA and siRNA pathways intersect is not well understood in most animals, but in *Caenorhabditis elegans* the two pathways are tightly connected (1,2). *Caenorhabditis elegans* piRNAs are 21 nucleotides (nt) long and contain a 5′ uracil (U) (4–7). Each piRNA is processed from its own autonomous transcript (8,9). piRNAs associate with the Piwi protein PRG-1 within the germline where they engage in mRNA surveillance (4–6). It is not known if piRNAs directly silence their targets; however, piRNAs act as a potent trigger for siRNA production from target mRNAs (6,10–14). Secondary siRNAs produced from piRNA targets are 22 nt long, contain a 5′ guanine (G) and associate with several Argonautes in the worm-specific WAGO subfamily, and are thus commonly called WAGO-class 22G-RNAs (15). WAGO-class 22G-RNA production is correlated with RNA silencing. Thus, piRNAs presumably orchestrate RNA silencing by triggering the production of WAGO-class 22G-RNAs from target mRNAs.

A second class of 22G-RNAs associates with the Argonaute CSR-1 and acts seemingly in opposition to piRNAs to promote germline gene expression (16–19). WAGO- and CSR-1-class 22G-RNAs share many of the same genetic requirements but differ in their dependency on *mutator* (*mut*) genes for their formation (15,16,20). WAGO-class 22G-RNAs are synthesized by an RNA-dependent RNA polymerase, which functions as part of a protein complex that is seeded by the intrinsically disordered protein MUT-16 at the cytoplasmic surface of the nuclear envelope in structures called *Mutator* foci (21). *Mutator* foci are adjacent to P granules, germ granules in which much of the piRNA machinery is housed. Thus, the WAGO-class 22G-RNA machinery and the piRNA machinery reside in close proximity to one another but appear to occupy largely distinct compartments (4,5,21).

The 22G-RNAs produced from piRNA targets can provide a molecular readout for piRNA activity (10,13). However, the presence of WAGO-class 22G-RNAs is not in and of itself indicative of an mRNA having been targeted by piRNAs, as there are other mechanisms that can trigger mRNA entry into the WAGO-class 22G-RNA pathway (22). Furthermore, in some instances, piRNAs initiate WAGO-class 22G-RNA production but are then dispensable for continued propagation of 22G-RNAs from an mRNA target, which can persist in the absence of the piRNA trigger for multiple generations (11,12,14). Recently, an improved understanding of piRNA-target recognition rules and biochemical experiments to identify PRG-1 interacting mRNAs have revealed that piRNAs interact with essentially all germline mRNAs (23,24). However, in addition to CSR-1-class 22G-RNAs, at least two other mechanisms exist, both involving cis-acting sequence elements, to counter piRNA-mediated gene silencing, and thus it is not clear to what extent piRNAs regulate germline gene expression (23,25). Neither piRNAs nor WAGO-class 22G-RNAs are essential for development at favorable growth temperatures but mutations in core factors in the pathways, such as *prg-1* or *mut-16*, respectively, cause reduced fertility that is exacerbated at higher temperatures (4–6,20).

Attempts to identify the roles of piRNAs in regulating gene expression on a genome-wide scale have been limited in their scope and confounded by whole animal-based approaches that fail to account for the diminished germlines of piRNA-defective mutants (4,5,10,13,26,27). Furthermore, genomic approaches to identify the roles of WAGO-class 22G-RNAs in regulating gene expression are also needed to better understand RNA silencing in the germline. Here we explore the roles of piRNAs and WAGO-class 22G-RNAs in regulating gene expression in the adult germline through parallel mRNA and small RNA sequencing from dissected gonads of *prg-1* and *mut-16* mutants. The results provide a comprehensive analysis of gene regulation by piRNAs and WAGO-class 22G-RNAs, revealing extensive roles for the two classes of small RNAs in shaping the germline transcriptome and uncovering a complex relationship between small RNAs and mRNA expression.

## MATERIALS AND METHODS

### Strains

NL1810[mut-16(pk710) I] (28) and SX922[prg-1(n4357) I] (6) were outcrossed to wild type (N2) 1× just prior to expansion for gonad dissections and RNA sequencing. DUP178[*glh-1(sam24[glh-1::gfp::3xFlag]) prg-1(sam97[TagRFP::3xFlag::PRG-1])* I] (29) and USC717[*mut-16(cmp3[mut-16::gfp::3xFLAG + loxP])* I] (30) were used to examine PRG-1 and MUT-16 expression in animals at the stage in which gonad dissections were done. TAM24[*mut-16(ram18[ko(302–4051])* I], containing a 3750 bp deletion in *mut-16*, and TAM22[*prg-1(ram17[ko(615–2575)])* I] containing a 1,961 bp deletion in *prg-1*, were generated using CRISPR-Cas9 genome editing (31–33). Double strand breaks were induced on both the 5′ and 3′ ends of the respective genes by introducing a Cas9 ribonucleoprotein complex containing IDT Alt-R crRNAs (TAM24: ACCCCACCAGAAACGAUAC and CAACCUGCUUAUAAUCACGU; TAM22: UACAAUAUGAGCAUCUUGCC and GGUUCCACAGUUCGUCAACC). Double strand breaks were presumably repaired through endogenous non-homologous end joining mechanisms. Candidates were screened for large deletions using PCR and Sanger sequencing. TAM40[*prg-1(ram22[D583A])* I] was generated by introducing a Cas9 ribonucleoprotein complex containing an IDT Alt-R crRNA (UACCACGACUCGACAUUGAA), resulting in a double strand break adjacent to the D583 residue of the DDH catalytic site. Double strand breaks were repaired from a single stranded donor oligonucleotide (IDT Ultramer DNA Oligo: CATTCCGCTTAAAAACACAATGATCGTCGGCTACGCTCTGTATCATGATTCAACATTGAAAGGAAAAACTGTCGGTGCTTGCGTGTC) which introduced a point mutation that converts the aspartic acid residue to alanine. Silent mutations were introduced into the donor oligonucleotide to prevent re-cutting at the locus. Candidates were screened using PCR and Sanger sequencing.

### Gonad dissections

Gonads were dissected from gravid adults grown at 20°C for 68–70 h post L1 synchronization as described (34). The proximal arms of the gonads were discarded such that only the distal arms were captured.

### RNA isolation

Whole animals and dissected distal gonads (∼500 gonads per replicate, three replicates per strain) were collected into Trizol, flash frozen in liquid nitrogen, thawed, and subjected to two chloroform extractions followed by isopropanol precipitation overnight at −80°C.

### mRNA-seq libraries

Total RNA was depleted of ribosomal RNA using the Ribo-Zero rRNA Removal Kit (Illumina). rRNA-depleted RNA was size selected (>200 nucleotides) to remove 5S rRNA and tRNA using RNA Clean & Concentrator-5 Kit (Zymo Research). Sequencing libraries were prepared using the NEBNext Ultra II Directional RNA Library Prep Kit for Illumina (NEB). All cDNA and PCR products were purified with AMPure XP beads. Samples were sequenced on an Illumina NextSeq 500 (High Output Kit, single-end, 75 cycles).

### mRNA-seq data analysis

Adapters and low-quality bases were removed from high-throughput sequencing reads using Trimmomatic v. 0.35 (35). Trimmed reads were mapped to the *C. elegans* genome (Wormbase release WS230) or transposon consensus sequences (36) using Star v. 2.5.0a (37). Reads from specific features were counted using RSEM v. 1.3.0 (38), except reads from transposon consensus sequences, which were counted with SAMtools (39). Differential expression analysis was done using DESeq2 v. 1.18.1 (40). In addition to the data reported here, RNA-seq libraries from *henn-1(pk2295)* mutant gonad samples were processed, normalized and analyzed in parallel and reported in Svendsen *et al.* (41). A 1.3 fold-change cutoff and a corrected *P*-value cutoff of 0.05 were applied when filtering for differentially expressed genes. Venn diagrams were drawn with BioVenn (42) and InteractiVenn (43). The plots modeled after UpSet plots were drawn in Adobe Illustrator (44). All other plots were drawn in R, Excel and IGV (45). See Supplementary Table S1 for additional details.

### Small RNA-seq libraries

16–30-nt RNAs were size selected on 17% polyacrylamide/urea gels. Purified small RNAs were treated with RNA polyphosphatase (Illumina) to reduce 5′ di- and triphosphates to monophosphates to enable 3′ adapter ligation to 22G-RNAs. Sequencing libraries were prepared using the NEBNext Multiplex Small RNA Library Prep Set for Illumina (NEB). PCR amplicons were size selected on 10% polyacrylamide gels. Samples were sequenced on an Illumina NextSeq 500 (High Output Kit, single-end, 75 cycles).

### Small RNA-seq data analysis

Small RNA sequences were parsed from adapters and trimmed reads with >1 base having a Phred quality score <30 were discarded. The remaining reads were mapped to the *C. elegans* genome (Wormbase release WS230) using CASHX v. 2.3 (46) or transposon consensus sequences (36) using Bowtie2 (47). Imperfectly matching reads were discarded. Reads from specific features were counted using custom Perl scripts and SAMtools (39). Small RNA features were classified as described (48). Differential expression analysis was done using DESeq2 v. 1.18.1 (40). In addition to the data reported here, RNA-seq libraries from *henn-1(pk2295)* mutant gonad samples were processed, normalized, and analyzed in parallel and reported in Svendsen *et al.* (41). A 1.3 fold-change cutoff and a corrected *P*-value cutoff of 0.05 were applied when filtering for differentially expressed small RNAs. Custom Perl and Python scripts, R, Excel and IGV were used for all other data analyses and for drawing plots. See Supplementary Table S1 for additional details. The HRDE-1 co-IP data analysis was described previously (49).

### Imaging

Adult stage *C. elegans* expressing GLH-1::GFP and RFP::PRG-1 or MUT-16:GFP were imaged on a Zeiss Axio Imager Z2 microscope after immobilization in 25 uM sodium azide.

### Quantitative RT-PCR

Total RNA from whole adult stage animals (72 h post L1 synchronization) was treated with Turbo DNase (ThermoFisher) and subjected to reverse transcription using SuperScript III (ThermoFisher) and random hexamer primers. qRT-PCR was done using iTaq Universal SYBR Green Supermix (Bio-Rad) and primers complementary to the *his-10* family, which also includes *his-14* and *his-26* (CATCCAAGGTATCACCAAGCCG and GTATGTGACGGCATCACGGATC), and the *his-12* family, which also includes *his-43* and *his-16* (CCCAAGACATCTTCAACTTGCC and CTCCTCCTTGAGCGATTGTG). Because of the similarity in histone genes, we cannot rule out that additional histones with near perfect complementarity to the primer sequences were not also amplified. Average Ct values were calculated for three biological replicates with 3–6 technical replicate PCRs done in parallel. Relative histone mRNA levels were calculated using the 2^−ddCt^ method (50). *rpl-32* levels were used for normalization.

### Statistical analysis

Benjamin-Hochberg corrected *P*-values are reported for all differential expression analysis. An arbitrary 1.3 fold-change and false discovery rate of 0.05 was applied when interpreting differentially expressed features, unless otherwise indicated. A hypergeometric test was used to assess statistical significance in the overlap of gene lists. Two-sample *t*-tests were used when comparing total mRNA or small RNA reads between different histone families and a Bonferroni correction was applied to account for multiple comparisons. *P*-values for qRT-PCR assays were calculated using Tukey HSD tests assessing all possible pairwise comparisons. Only *P*-values for relevant comparisons are reported.

## RESULTS

### High-throughput sequencing of mRNAs and small RNAs from adult gonads

**Figure 1.**
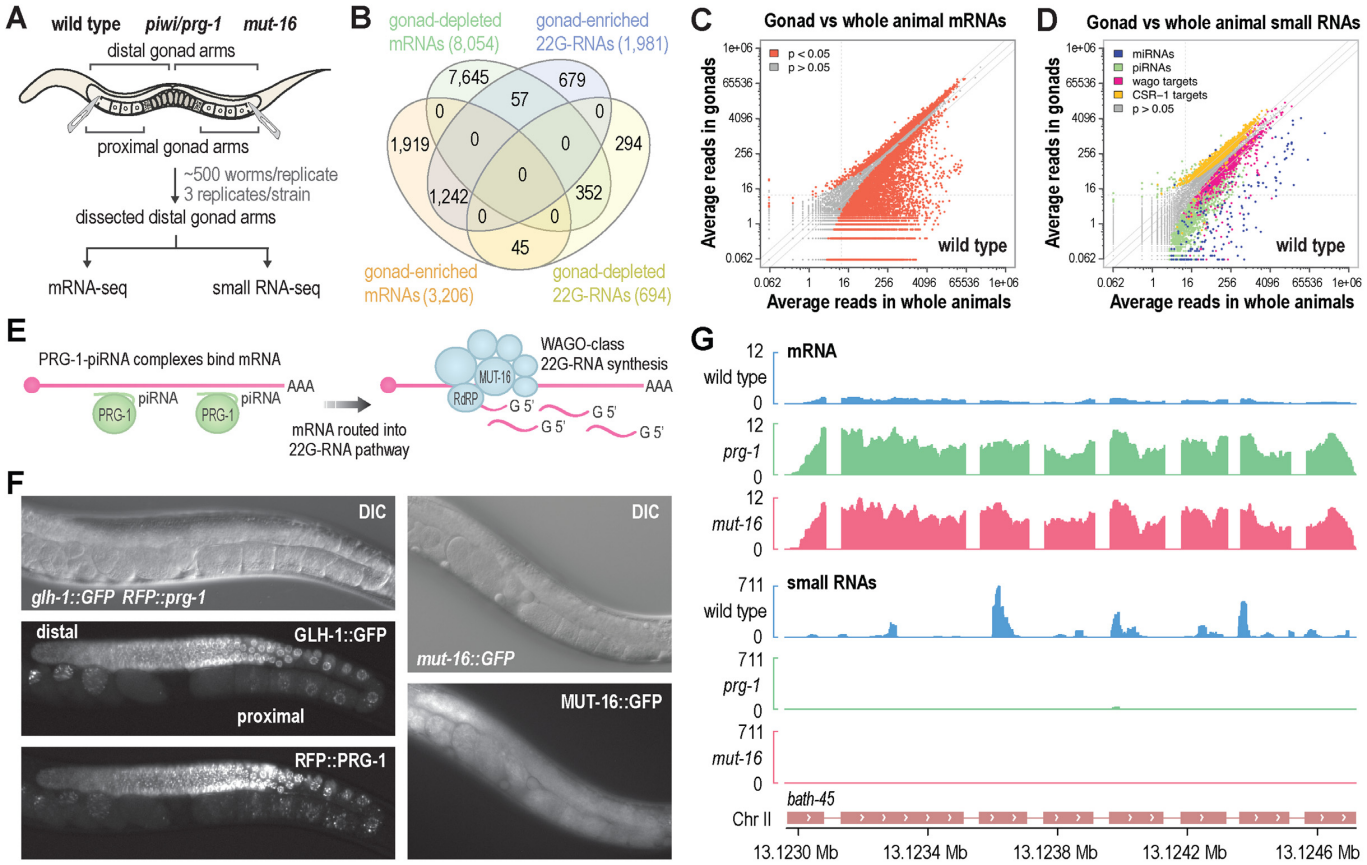
Small RNA and mRNA sequencing on whole animals and dissected gonads. (**A**) Gonads were dissected and proximal gonad arms removed from wild type animals and *prg-1(n4357)* and *mut-16(pk710)* mutants. RNA from distal gonad arms, as well as a subpopulation of wild type whole animals, was subjected to small RNA and mRNA high-throughput sequencing. (**B**) Overlap between mRNAs and 22G-RNAs enriched in distal gonads or whole animals based on a corrected p-value cutoff of 0.05 and a 1.3-fold change cutoff. (**C**) Scatter plot displaying each mRNA as a function of average normalized reads in gonads (y-axis) versus whole animals (x-axis) (*n* = 3 biological replicates). (**D**) Scatterplot displaying each small RNA feature (miRNA, piRNA, WAGO-class 22G-RNA locus, and CSR-1-class 22G-RNA locus) as a function of average normalized reads in distal gonads (y-axis) versus whole animals (x-axis) (*n* = 3 biological replicates). (**E**) Model showing piRNAs bound to Piwi/PRG-1 directing their mRNA targets into the RNAi pathway in which an RNA-dependent RNA polymerase, in a complex with MUT-16 and other mutator proteins, synthesizes 22G-RNAs antisense to the mRNA target that will go on to bind WAGO subfamily Argonautes. (**F**) RFP::PRG-1 and MUT-16::GFP expression in adult animals at the same age as the animals used in the gonad dissections illustrated in (A). GLH-1::GFP is shown as a germ cell marker. The distal and proximal gonad arms are indicated. (**G**) mRNA and small RNA read distribution across a well-characterized piRNA and 22G-RNA target gene, *bath-45*, in wild type animals and *prg-1(n4357)* and *mut-16(pk710)* mutants. For simplicity, strandedness is not shown.

piRNAs and WAGO-class 22G-RNAs are both required for optimal fertility but their impact on endogenous mRNA expression is not well understood (4–6,20). To explore the roles of piRNAs and WAGO-class 22G-RNAs in regulating gene expression in the *C. elegans* germline, we isolated RNA from gonads dissected from adult wild type animals and from *prg-1(n4357)* and *mut-16(pk710)* mutants. Our samples contained the distal arms of the gonad that are comprised of both mitotic and meiotic germ cells but excluded the proximal arms that contain the oocytes and sperm (Figure 1A). Total RNA >200 nt long was depleted of ribosomal RNAs and subjected to high-throughput sequencing. In parallel, we also sequenced 16–30 nt small RNAs. To categorize mRNAs and small RNAs enriched or depleted in the dissected distal gonad arms relative to whole animals, we also subjected RNA from a subpopulation of our wild type whole animals to RNA-seq (Supplementary Table S1).

We then compared gene expression in our wild type gonad and whole animal libraries to identify mRNAs and small RNAs predominantly expressed in the distal germline. An arbitrary false discovery rate of 0.05 was applied for reporting misregulated genes throughout this study. Additionally, a 1.3-fold-change cutoff was applied when reporting differentially expressed small RNAs and mRNAs, which excluded many misregulated genes based on a *P*-value cutoff of 0.05 but is more likely to reflect biologically relevant changes in expression. We identified 3206 annotated mRNAs and 1981 annotated 22G-RNA loci enriched in our distal gonad libraries, of which 1242 corresponded to a common set of genes (Figure 1B–D and Supplementary Tables S2 and S3). 8054 mRNAs were reduced in our distal gonad libraries relative to whole animals and are thus predominantly expressed in the soma or gametes (Figure 1B and C and Supplementary Table S4). The majority of miRNAs (181), and many WAGO-class 22G-RNAs (672) and piRNAs (1523), were depleted in the distal gonad samples, indicating that they are preferentially expressed in either somatic or gametic cells (Figure 1D and Supplementary Table S5). Given that piRNAs are primarily expressed in germ cells, it is likely that those that were depleted in distal gonads tend to be expressed more highly in sperm and oocytes. The vast majority (∼95%) of small RNAs enriched in the distal gonad libraries were CSR-1 class 22G-RNAs, indicating that their expression is highest in non-gametic germ cells (Figure 1D and Supplementary Table S3).

These datasets enable parallel analysis of small RNA and mRNA expression in the distal gonad, thereby establishing a valuable framework for exploring the roles of small RNAs in gene regulation in the distal germline. The data can be visualized in Integrative Genomics Viewer and is available for download as a user-friendly standalone session at https://www.montgomerylab.org/resources.html (51).

### Gonad-seq on *prg-1* and *mut-16* mutants

PRG-1 is the only known binding partner of piRNAs in *C. elegans*, and in *prg-1* mutants, piRNAs are lost (4–6). Mutations in *mut-16*, a gene required for the formation of the RNA-dependent RNA polymerase complex that synthesizes 22G-RNAs, abolish WAGO-class 22G-RNA production downstream of piRNAs and other primary small RNAs (Figure 1E) (20,21). *prg-1* was expressed throughout the germline at the stage in which we collected animals for gonad dissections and displayed an almost identical expression pattern to that of *glh-1*, a major P granule component and germ cell marker (Figure 1F) (29). *mut-16* was also expressed throughout the gonad but was not obviously enriched in the germline relative to somatic tissues, consistent with its presumably ubiquitous role in RNAi and WAGO-class 22G-RNA pathways (Figure 1F) (20,21,30). Using our RNA-seq datasets from *prg-1* and *mut-16* mutant distal gonads, we assessed the roles of piRNAs and WAGO-class 22G-RNAs in regulating gene expression in the distal germline. As proof of principle, we examined small RNA and mRNA read distribution across *bath-45*, a relatively well characterized piRNA target that produces high levels of WAGO-class 22G-RNAs (10,11,13). Consistent with previous studies, *bath-45*-derived 22G-RNAs were lost in *prg-1* and *mut-16* mutants, whereas mRNA levels were upregulated ∼10-fold (Figure 1G). Thus, our data faithfully reflects previous studies, thereby enabling us to assess more broadly the roles of piRNAs and WAGO-class 22G-RNAs in regulating gene expression in the distal germline. The *prg-1* and *mut-16* datasets used in this study are also available for download and visualization at https://www.montgomerylab.org/resources.html.

### Widespread gene misexpression in the distal gonads of *prg-1* and *mut-16* mutants

**Figure 2.**
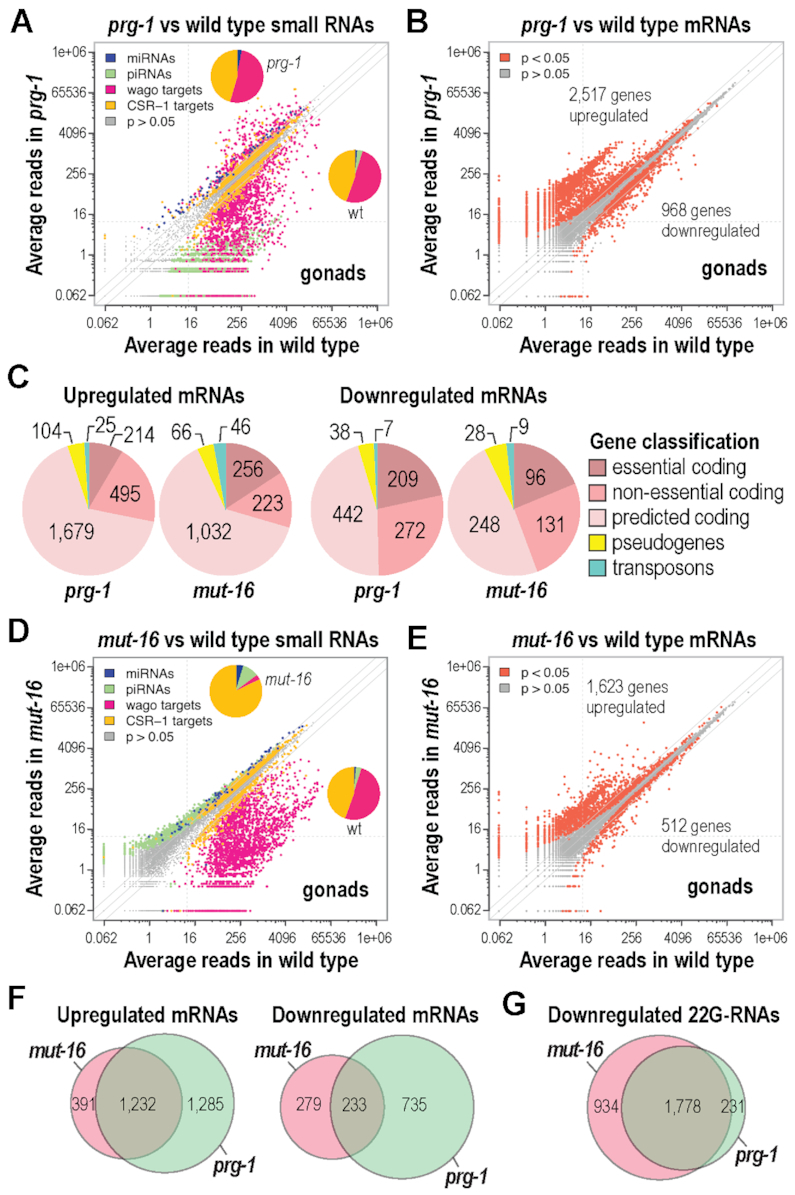
High-throughput sequencing of mRNAs and small RNAs from the distal gonads of *prg-1* and *mut-16* mutants. (**A**) Scatterplot displaying each small RNA feature (miRNA, piRNA, WAGO-class 22G-RNA locus, and CSR-1-class 22G-RNA locus) in *prg-1(n4357)* (y-axis) versus wild type (x-axis). Inset pie charts display the proportion of each class of small RNAs within each library. (**B**) Scatterplot displaying each mRNA as a function of average normalized reads in *prg-1(n4357)* (y-axis) versus wild type (x-axis). The numbers of genes misexpressed are shown. (**C**) Pie charts showing the classification of mRNAs differentially expressed (*P* < 0.05, fold-change > 1.3) in *prg-1(n4357)* and *mut-16(pk710)* mutants. (**D**) Scatterplot displaying each small RNA feature, as in (A), in *mut-16(pk710)* (y-axis) versus wild type (x-axis). Inset pie charts display the proportion of each class of small RNAs within each library. (**E**) Scatterplot displaying each mRNA as a function of average normalized reads in *mut-16(pk710)* (y-axis) versus wild type (x-axis). The numbers of genes misexpressed are shown. (**F**) Overlap in upregulated and downregulated mRNAs (*P* < 0.05, fold-change > 1.3) between *prg-1(n4357)* and *mut-16(pk710)* mutants. (**G**) Overlap in downregulated 22G-RNAs (*P* < 0.05, fold-change > 1.3) between *prg-1(n4357)* and *mut-16(pk710)* mutants.

We first did a general analysis of small RNA and mRNA misexpression in the distal gonads of piRNA and WAGO-class 22G-RNA defective mutants, focusing initially on *prg-1* and the piRNA pathway. In *prg-1(n4357)* mutants, ∼66% of annotated WAGO targets were depleted of 22G-RNAs and nearly all piRNAs were lost, consistent with whole animal studies (Figure 2A and Supplementary Tables S6 and S7) (10,13). Within our mRNA sequencing datasets, 2517 genes were upregulated and 968 genes were downregulated in *prg-1* mutants relative to wild type after applying an arbitrary 1.3-fold-change cutoff (*P* < 0.05) (Figure 2B and Supplementary Tables S8 and S9). By extension, ∼26% of the 13 367 distal germline expressed genes (mRNAs we captured with a base mean number of reads > 1) were misregulated in *prg-1* mutants. Among the differentially expressed genes, the majority corresponded to predicted or validated coding genes, many of which are annotated as causing lethality or sterility when knocked down or mutated (Figure 2C). These results point to broad roles for piRNAs in shaping the germline transcriptome and suggest that their functions extend far beyond their well-known roles in silencing non-self and aberrant genes.

We then assessed the role of *mut-16* and thus the WAGO-class 22G-RNA pathway in regulating gene expression in the distal gonad. As predicted based on previous studies exploring small RNA expression in whole animals, WAGO-class 22G-RNAs were strongly depleted in *mut-16* mutants (Figure 2D and Supplementary Tables S10 and S11) (20,21,52). However, there was also a modest reduction in 22G-RNAs levels for 341 CSR-1 target genes, possibly because of competition between the WAGO-class Argonautes and CSR-1 such that some mRNAs are targeted by both pathways (Figure 2D and Supplementary Table S10) (52). In our mRNA-sequencing libraries, we identified 1623 genes upregulated and 512 genes downregulated >1.3-fold in distal gonads dissected from *mut-16(pk710)* mutants relative to wild type gonads (Figure 2E and Supplementary Tables S12 and S13). Similar to *prg-1* mutants, most genes misexpressed in *mut-16* mutants are annotated as protein coding genes and many are annotated as being essential for survival or fertility (Figure 2C). We conclude that, like piRNAs, WAGO-class 22G-RNAs have widespread roles in regulating gene expression in the germline.

Next we compared the overlap in mRNAs and small RNAs misexpressed in *prg-1* and *mut-16* mutants. Because piRNAs trigger WAGO-class 22G-RNA production from target mRNAs, we predicted similar effects on gene expression in *prg-1* and *mut-16* mutants. Indeed, there was considerable overlap in the mRNAs upregulated or, to a lesser degree, downregulated in *prg-1* and *mut-16* mutants, although many genes were uniquely affected in one strain or the other (Figure 2F). It is not unexpected that mutations in *mut-16* would affect a subset of mRNAs not affected by *prg-1*, as WAGO-class 22G-RNA production can be triggered through piRNA-independent mechanisms (22). However, it is surprising that ∼60% more mRNAs were misregulated in *prg-1* mutants than in *mut-16* mutants, given that piRNAs are thought to function exclusively through the WAGO-class 22G-RNA pathway (10,13). It is possible that piRNAs function in two distinct modes, one of which is not dependent on the WAGO-class 22G-RNA pathway for target regulation. Nonetheless, consistent with the characterized role of piRNAs in triggering WAGO-class 22G-RNA production, ∼89% of loci depleted of 22G-RNAs in *prg-1* mutants were also depleted in *mut-16* (Figure 2G).

We were not able to identify any high-confidence features uniquely associated with the genes specifically upregulated in only one of the two strains. However, ∼10% of the genes uniquely upregulated in *mut-16* mutants are annotated as transposons suggesting that *mut-16* may be more broadly required for transposon silencing than *prg-1*. Several of the genes uniquely downregulated in *prg-1* are associated with P granule assembly or function, including *glh-2*, *meg-1*, *meg-2*, *mex-1* and *mes-1* (Supplementary Table S9). Additionally, many histone genes were strongly downregulated in *prg-1* mutants, which we did not observe to the same extent in *mut-16* mutants, although there was a modest reduction (<1.7-fold) in some histone mRNA levels in *mut-16* (Supplementary Tables S9 and S13). In the following sections, we explore the common and unique roles for *prg-1* and *mut-16* in regulating gene expression in the distal germline.

### Misregulation of spermatogenic and oogenic genes in *prg-1* and *mut-16* mutants

**Figure 3.**
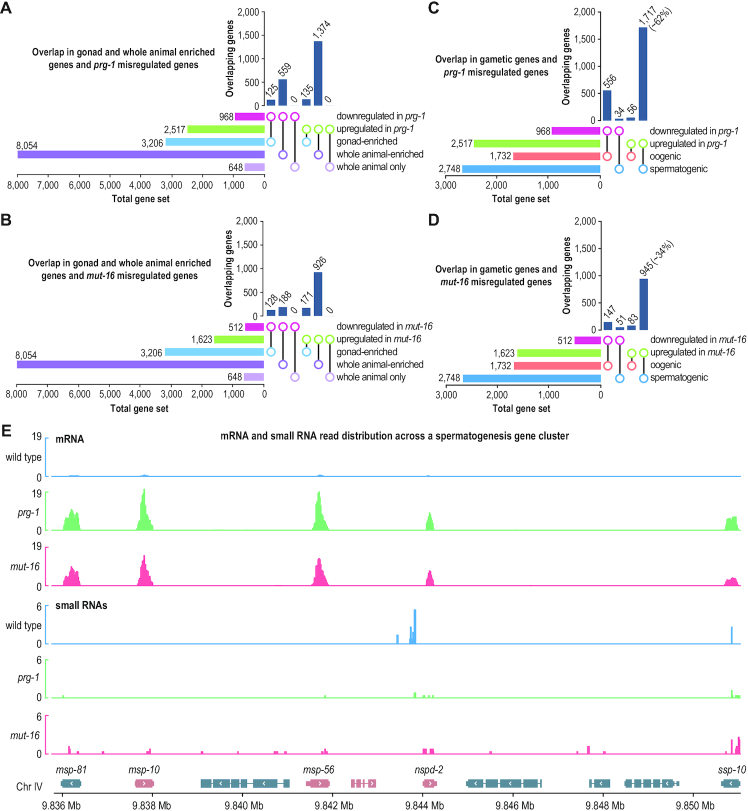
Misregulation of gametic genes in *prg-1* and *mut-16* mutant gonads. (**A**, **B**) UpSet plots displaying the overlap in mRNAs upregulated or downregulated in *prg-1(n4357)* (A) and *mut-16(pk710)* (B) mutants and mRNAs enriched in whole animals or dissected distal gonads (*P* < 0.05, fold-change > 1.3). (**C-D**) UpSet plots displaying the overlap in mRNAs upregulated or downregulated (*P* < 0.05, fold-change > 1.3) in *prg-1(n4357)* (C) and *mut-16(pk710)* (D) mutants and mRNAs enriched in spermatogenic or oogenic gonads. The percentages shown are for the gene sets upregulated in *prg-1* or *mut-16* mutants. (**E**) mRNA and small RNA read distribution across a cluster of spermatogenesis genes (gene names shown only for sperm genes) in wild type animals and *prg-1(n4357)* and *mut-16(pk710)* mutants. For simplicity, strandedness is not shown.

To identify common roles for the piRNA and WAGO-class 22G-RNA pathways, we examined the genes misexpressed in both *prg-1* and *mut-16* mutants (Figure 2F). Most mRNAs misexpressed in either *prg-1* or *mut-16* mutants were depleted in our wild type libraries from distal gonads, which, as noted above, are comprised primarily of germ cells but lack sperm and oocytes, and were enriched in our whole animal libraries (Figure 3A and B). This suggests that genes misregulated in *prg-1* and *mut-16* are preferentially expressed in the proximal gonad or in somatic cells.

PRG-1 and MUT-16 localize at or adjacent to P granules, ribonucleoprotein compartments that reside on the cytoplasmic surface of germ cell nuclei (4,5,21). P granules are implicated in silencing somatic genes in the germline and consequently it is possible that piRNAs and WAGO-class 22G-RNAs mediate somatic gene silencing (53,54). However, while most mRNAs misexpressed in the distal gonads of *prg-1* and *mut-16* mutants were enriched for expression in whole animals relative to gonads, none of the mRNAs that were expressed exclusively in whole animals and not in gonads, were misregulated in either *prg-1* or *mut-16* mutants (Figure 3A and B and Supplementary Table S14). Additionally, there was very little overlap between the mRNAs misregulated in *prg-1* (∼7% overlap) or *mut-16* (∼6% overlap) mutants and the 1181 mRNAs previously classified as soma-specific by Knutson *et al.* (53). Instead, the majority of mRNAs misregulated in *prg-1* and *mut-16* mutant distal gonads were amongst the top 6,000 mRNAs captured in sperm or oocyte RNA-seq libraries (Supplementary Figure S1) (55,56). For example, ∼64% of mRNAs downregulated in *prg-1* mutants were amongst the top 6,000 expressed in oocytes and ∼56% of mRNAs upregulated in *prg-1* mutants were amongst the top 6000 expressed in sperm (Supplementary Figure S1A). Similarly, ∼46% of mRNAs downregulated in *mut-16* mutants were amongst the top 6,000 oocyte-expressed genes, whereas ∼53% of mRNAs upregulated in *mut-16* mutants were amongst the top 6000 sperm-expressed genes (Supplementary Figure S1A). Thus, it is likely that the genes misregulated in *prg-1* and *mut-16* mutants are predominantly expressed in gametes rather than in somatic cells, indicating that other factors contribute to P granule-mediated silencing of somatic genes.

The elevated levels of genes expressed in sperm and the reduced levels of genes expressed in oocytes in the distal gonads of *prg-1* and *mut-16* mutants points to a possible role for *prg-1* and *mut-16* in regulating spermatogenesis and oogenesis. Therefore, to assess the role of piRNAs and WAGO-class 22G-RNAs in regulating spermatogenic and oogenic genes, we compared the mRNAs misregulated in our distal gonad libraries from *prg-1* and *mut-16* mutants with mRNAs enriched in oogenic or spermatogenic gonads (57). There was a slight underrepresentation in the 1732 genes enriched in oogenic gonads within our datasets of mRNAs upregulated in *prg-1* (∼3.3-fold underrepresentation, *P* < 0.0005) and *mut-16* (∼1.7-fold underrepresentation, *P* < 0.0005) relative to what would be expected by chance (Figure 3C and D). In contrast, there was overrepresentation of oogenic genes within our datasets of mRNAs downregulated in *prg-1* (∼7-fold enrichment, *P* < 0.0005) and *mut-16* (∼3-fold enrichment, *P* < 0.0005) (Figure 3C and D). Of the 2748 mRNAs enriched in spermatogenic gonads, ∼62% were upregulated in *prg-1* mutants (∼5-fold overrepresentation, *P* < 0.0005) and ∼34% were upregulated in *mut-16* mutants (∼4-fold overrepresentation, *P* < 0.0005) (Figure 3C and D). The median fold change in mRNAs upregulated in spermatogenic gonads was ∼22-fold in *prg-1* mutants and ∼8-fold in *mut-16* mutants, as illustrated by a cluster of spermatogenesis genes on chromosome II (Figure 3E). Not surprisingly, *bath-45*, the piRNA target described above (Figure 1G), is also enriched in the spermatogenic gonad (57).

Gonads in this study were dissected from adult animals, at which time the hermaphroditic germline has normally fully transitioned from spermatogenesis to oogenesis. The upregulation of spermatogenic genes and downregulation of oogenic genes we observed is consistent with tiling array experiments involving whole adult *prg-1* mutants (5) and suggests that *prg-1* and *mut-16* mutants may be defective in transitioning from spermatogenesis to oogenesis. To assess whether the effect on spermatogenic genes is directly related to 22G-RNA expression, we examined the relationship between the spermatogenic mRNAs upregulated or downregulated in *prg-1* and *mut-16* mutants and changes in 22G-RNA levels from these genes. There was a tendency for spermatogenic mRNAs upregulated in either *prg-1* or *mut-16* to also have altered levels of 22G-RNAs (Supplementary Figure S2). However, ∼48% (824) of spermatogenic mRNAs upregulated in *prg-1* mutants and ∼63% (596) upregulated in *mut-16* mutants did not have detectable changes in 22G-RNA levels (Supplementary Figure S2). Many in fact had elevated levels of 22G-RNAs, contrary to what would be predicted if these mRNAs were directly regulated by piRNAs or WAGO-class 22G-RNAs (Supplementary Figure S2). This suggests that the impact of piRNAs and WAGO-class 22G-RNAs on gametic gene expression is at least partially indirect and may be caused by defects in cell specification or other abnormalities in the germlines of *prg-1* and *mut-16* mutants. It is also possible that mutations in *prg-1* and *mut-16* shift the balance away from WAGO-class 22G-RNAs towards the production of CSR-1-class 22G-RNAs. This may explain why large proportions of spermatogenic genes, particularly those upregulated in *prg-1* mutants, have elevated levels of 22G-RNAs in *prg-1* and *mut-16* mutants.

### Transposon desilencing in *prg-1* and *mut-16* mutants

**Figure 4.**
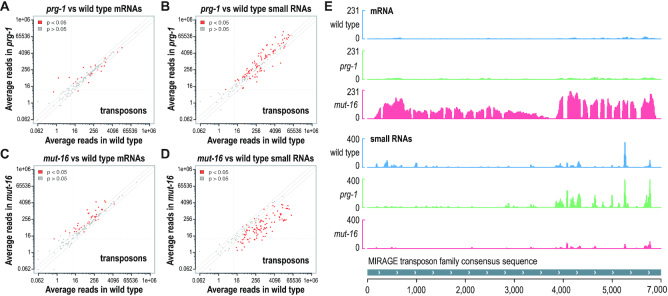
Transposon misexpression in *prg-1* and *mut-16* mutants. (**A**) Each transposon family (152 total) is plotted as a function of mRNA reads in *prg-1(n4357)* (y-axis) versus wild type (x-axis) distal gonads. (**B**) Each transposon family is plotted as a function of small RNA reads in *prg-1(n4357)* (y-axis) versus wild type (x-axis) gonads. (**C**) Each transposon family (152 total) is plotted as a function of mRNA reads in *mut-16(pk710)* (y-axis) versus wild type (x-axis) gonads. (**D**) Each transposon family is plotted as a function of small RNA reads in *mut-16(pk710)* (y-axis) versus wild type (x-axis) gonads. (**E**) mRNA and small RNA read distribution across the MIRAGE1 transposon consensus sequence in wild type animals and *prg-1(n4357)* and *mut-16(pk710)* mutants. For simplicity, strandedness is not shown.

piRNAs and siRNAs are well known for their roles in silencing transposons (1). However, in *C. elegans*, the extent to which piRNAs and siRNAs impact transposon expression is not clear. To explore the roles of piRNAs and WAGO-class 22G-RNAs in regulating transposons, we extracted reads mapping to each of the 152 transposon family consensus sequences within our mRNA and small RNA sequencing datasets from distal gonads of *prg-1* and *mut-16* mutants (36). Of the 152 transposon families, only 11 were upregulated >1.3-fold in *prg-1* mutants, only one of which was depleted of 22G-RNAs (Figure 4A and Supplementary Table S15). Furthermore, only 21 transposon families were depleted of 22G-RNAs in *prg-1* mutants, whereas 72 had elevated levels of 22G-RNAs, the reason for which is unclear (Figure 4B and Supplementary Table S15). In contrast, 34 transposon families had elevated mRNA levels in *mut-16* mutants, 30 of which were depleted of 22G-RNAs in *mut-16* mutants and are thus direct targets of the WAGO-class 22G-RNA pathway (Figure 4C and Supplementary Table S16). 22G-RNAs from 101 transposon families were depleted in *mut-16* mutants, however, the corresponding mRNAs were upregulated >1.3-fold in only 30 of these, suggesting that loss of 22G-RNAs from most transposon families has little impact on their expression (Figure 4C and D and Supplementary Table S16).

The fertility defects in both *prg-1* and *mut-16* mutants are exacerbated at 25°C (4,5,20). Although it is not clear what causes the loss of fertility at 25°C, it is possible that elevated levels of transposon transposition is responsible. If so, we would predict that transposon mRNA levels would be elevated at 25°C relative to 20°C. To test this, we again sequenced mRNAs from wild type animals and *prg-1(n4357)* and *mut-16(pk710)* mutants, this time using whole adult animals grown at 20°C or 25°C. Surprisingly, there was very little difference in transposon misregulation in either *prg-1* or *mut-16* mutants when grown at 25°C versus 20°C, although we did observed modest differences in which transposons were affected (Supplementary Figure S3A–D and Supplementary Tables S15 and S16). This suggests that transposon misregulation is not responsible for the additional reduction in fertility that occurs in *prg-1* or *mut-16* mutants when grown at 25°C compared with 20°C.

The Tc3 and MIRAGE transposon families were previously shown to be upregulated in *prg-1* mutants (5,6,26). We observed an ∼1.7-fold increase in Tc3 levels in *prg-1* mutants, similar to what was previously reported for this allele using quantitative RT-PCR, but substantially lower than the ∼3-4-fold upregulation observed in other *prg-1* alleles (Supplementary Figure S3E) (6). MIRAGE mRNA levels were upregulated ∼1.2-fold in *prg-1* mutants, which is below the 1.3 fold-change threshold we used for classifying differentially expressed genes, and substantially less than was previously shown in RNA-seq experiments using the same allele (Figure 4E) (26). However, both Tc3 and MIRAGE mRNA levels were upregulated ∼4–15-fold in *mut-16* mutants (Figure 4E and Supplementary Figure S3E).

Based on these results, we conclude that, in contrast to *mut-16* and the WAGO pathway, *prg-1* and the piRNA pathway have a modest role in maintaining transposon silencing in the distal germline, although it is possible that piRNAs have a role initiating transposon silencing that is maintained in the absence of *prg-1*. Alternatively, other features of transposons may direct their entry into the WAGO-class 22G-RNA pathway. Our results are consistent with a recent study showing that the frequency of transposon-induced double-strand breaks is much higher in *mut-16* mutants than in *prg-1* mutants (58).

### Histone misexpression in *prg-1* and *mut-16* mutants

**Figure 5.**
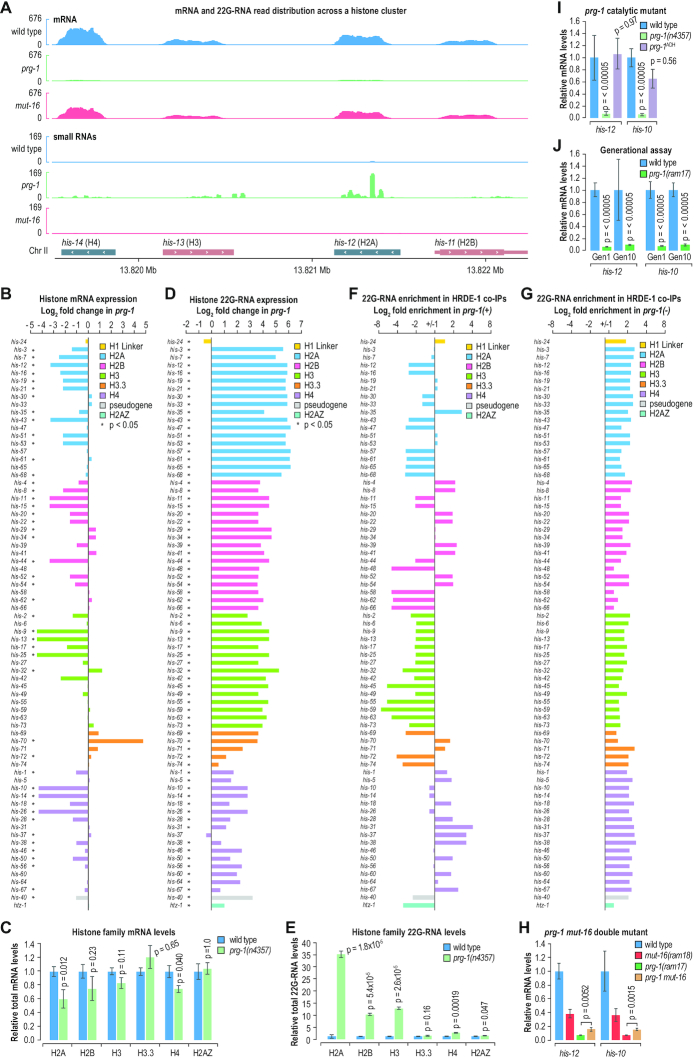
Histone misexpression in *prg-1* mutants. (**A**) mRNA and small RNA read distribution across a cluster of core histone genes in the distal gonads of wild type animals and *prg-1(n4357)* and *mut-16(pk710)* mutants. For simplicity, strandedness is not shown. (**B**) Bar plot displaying each histone gene as a function of its log_2_ fold-change in mRNA expression in *prg-1(n4357)* mutants relative to wild type distal gonads. Bars are colored by histone family as indicated in the key. (**C**) Total histone family mRNA levels in *prg-1(n4357)* mutants relative to wild type distal gonads. Error bars show standard deviation (*n* = 3 biological replicates). (**D**) Bar plot displaying each histone gene as a function of its log_2_ fold-change in 22G-RNA expression in *prg-1(n4357)* mutants relative to wild type distal gonads. Bars are colored by histone family as indicated in the key. (**E**) Total histone family small RNA levels in *prg-1(n4357)* mutants relative to wild type distal gonads. Error bars show standard deviation (*n* = 3 biological replicates). (**F-G**) Histone-derived 22G-RNA enrichment in FLAG::HRDE-1 co-IPs relative to input cell lysates from whole animals wild type (*prg-1(+)*) (F) or mutant (*prg-1(−)*) (G) for *prg-1*. The *prg-1* mutant allele is n4357. (**H**) qRT-PCR assay of *his-12* and *his-10* expression in wild type whole animals and *prg-1(ram17)* and *mut-16(ram18)* single and double mutants. Error bars show standard deviation (*n* = 3 biological replicates). (**I**) qRT-PCR assay of *his-12* and *his-10* expression in wild type whole animals and *prg-1(n4357)* and *prg-1*^*ADH*(ram22)^ mutants. Error bars show standard deviation (*n* = 3 biological replicates). (**J**) qRT-PCR assay of *his-12* and *his-10* expression in wild type whole animals and *prg-1(ram17)* mutants at one generation of growth directly after generating the line and again at 10 generations. Error bars show standard deviation (n = 3 biological replicates).

We next explored the roles of piRNAs and WAGO-class 22G-RNAs in regulating histone expression. Several histones were among the most highly downregulated genes in the distal gonads of *prg-1* mutants (Supplementary Table S9). For example, histones within the chromosome II cluster, which contains representatives from each of the four core histone families, were downregulated ∼10–20-fold in *prg-1* mutants (Figure 5A and Supplementary Table S9). The majority of the 65 canonical replication-dependent histone genes, corresponding to H2A, H2B, H3 and H4, were downregulated in *prg-1* mutants, although some of the core histone mRNAs were unchanged or upregulated in *prg-1* mutants (Figure 5B). However, summing total mRNA reads for each histone family, only H2A and H4 families were downregulated at a Bonferroni corrected p-value cutoff of 0.05 in *prg-1* mutants (Figure 5C). Coincident with the downregulation of histone mRNA levels was a dramatic increase in 22G-RNAs from histone genes, although most are not annotated as 22G-RNA loci (Figure 5A and D). With only two exceptions, 22G-RNA production from histone mRNAs was upregulated ∼1.5–73-fold in *prg-1* mutants relative to wild type animals (Figure 5D). Total 22G-RNA levels from H2A, the most strongly downregulated histone family at the mRNA level, were upregulated ∼35-fold in *prg-1* mutants (Figure 5E). H2B- and H3-derived 22G-RNAs were also upregulated >10-fold in *prg-1* mutants (Figure 5E). In contrast to the core replication-dependent histone mRNAs, the H1 linker histone and the replication-independent histone variants H3.3 and H2A.Z were not downregulated, and in some instances were upregulated, in *prg-1* mutants (Figure 5B).

Histone mRNA levels were only modestly affected in *mut-16* mutants and there was not a clear trend in upregulated and downregulated genes (Figure 5A and Supplementary Figure S4A). Total mRNA levels from each of the core histone families were unchanged in *mut-16* mutants (Supplementary Figure S4B). Furthermore, there was only a modest and inconsistent effect on histone-derived 22G-RNA levels in *mut-16* mutants (Supplementary Figure S4C and D). However, 22G-RNAs derived from *his-24*, an H1 linker histone, which is not one of the core histones and which ranks 14 among all genes in terms of total siRNAs produced in wild type animals, were depleted ∼93-fold in *mut-16* mutants, indicating that it is likely a *bona fide* target of the WAGO pathway (Supplementary Figure S4C). mRNA and 22G-RNA levels from the other H1-like genes, *hil-1-hil-8*, which are not well characterized, were only modestly affected or unchanged in *mut-16* mutants (Supplementary Tables S10–S13). Several other histones, particularly H4 family members, were also depleted of 22G-RNAs in *mut-16* mutants, suggesting that the WAGO pathway may have a role in suppressing certain histone genes, consistent with the modest upregulation of some histone mRNAs in *mut-16* mutants (Supplementary Figure S4A and C).

### Histone mRNAs are misdirected into the HRDE-1 nuclear RNAi pathway in *prg-1* mutants

Because most histone small RNA and mRNA levels were only modestly affected or unchanged in *mut-16* mutants, WAGO-class 22G-RNAs likely have a minor role in regulating histone genes under normal conditions. In contrast, the upregulation of 22G-RNAs derived from histone mRNAs and the reduction in histone mRNA levels in *prg-1* mutants suggests a prominent role for piRNAs in protecting histone mRNAs from RNA silencing. If so, we would predict that histone-derived 22G-RNAs interact with the downstream silencing machinery upon loss of piRNAs. We therefore tested whether the 22G-RNAs produced from histones associate with HRDE-1, a nuclear WAGO Argonaute that binds WAGO-class 22G-RNAs and promotes transgenerational inheritance of piRNA-mediated gene silencing (10–12,14,59). To examine histone-derived 22G-RNA association with HRDE-1, we compared 22G-RNA enrichment in FLAG::HRDE-1 co-immunoprecipitates (co-IPs) from whole animals wild type (+) or mutant (–) for *prg-1* (49). With the exception of the subset of histones depleted of 22G-RNAs in *mut-16* mutants (Supplementary Figure S4C), 22G-RNAs from most histones were underrepresented in FLAG::HRDE-1 co-IPs relative to cell lysates in *prg-1(*+*)* animals, indicating that they are not normally routed into the HRDE-1 pathway (Figure 5F). In contrast, in *prg-1(–)* animals, 22G-RNAs from each of the histone mRNAs were enriched in FLAG::HRDE-1 co-IPs (Figure 5G). This suggests that PRG-1 somehow prevents histone mRNAs from inappropriately entering the HRDE-1 nuclear RNAi pathway.

If *mut-16* and the WAGO-class 22G-RNA pathway are required for the histone silencing we observed in *prg-1* mutants, histone gene expression should be at least partially restored in *prg-1 mut-16* double mutants. To test this, we did qRT-PCR to test histone gene expression in a series of *prg-1* and *mut-16* single and double mutant strains we generated using CRISPR-Cas9 to delete the coding regions of the two genes. With these newly generated alleles, we could simultaneously confirm that the histone silencing phenotype was not related to background mutations in the *prg-1(n4357)* strain used in our RNA-seq experiments. Consistent with our RNA-seq results using the *prg-1(n4357)* mutant, the two histone genes we analyzed by qRT-PCR, *his-12* (H2A) and *his-10* (H4), were downregulated ∼14–15-fold in the *prg-1* deletion mutant (*P* < 0.0005) (Figure 5H). In the *prg-1 mut-16* double mutant, we observed an ∼2.5-fold increase in *his-12* and *his-10* expression relative to the *prg-1* single mutant (*P*-values < 0.01) (Figure 5H). However, *his-12* and *his-10* expression was still lower in the *prg-1 mut-16* double mutant than in the *mut-16* single mutant, indicating that inactivating *mut-16* only partially rescues histone expression in *prg-1* mutants (Figure 5H). Together, these results indicate that *prg-1* protects histones from silencing by the WAGO-class 22G-RNA pathway, but also that other factors contribute to the strong loss of histone expression in *prg-1* mutants and the modest loss in *mut-16* mutants.

### PRG-1 is not directly involved in histone 3′ end cleavage

The histones silenced in *prg-1* mutants are predominantly canonical replication-dependent histones, which are unusual in that they lack poly(A) tails and instead contain a hairpin in their 3′UTRs that promotes cleavage and 3′ end maturation (60). It is unclear how 3′ end maturation occurs in *C. elegans* as the U7 snRNA involved in cleaving histones in most metazoans is not found in worms (60). It is possible that PRG-1 promotes 3′ end cleavage and in its absence histone mRNAs are recognized as aberrant and thus routed into the HRDE-1 pathway for silencing. PRG-1 contains the catalytic triad of amino acid residues implicated in slicer activity and it is possible that PRG-1 cleaves histone mRNAs in place of the U7 associated machinery found in other metazoans (61). To test this, we introduced a mutation in one of the conserved catalytic residues of *prg-1* using CRISPR-Cas9. We then tested whether *his-12* and *his-10* were silenced in the *prg-1* catalytic mutant using qRT-PCR. We did not detect a difference in the levels of *his-12* or *his-10* in *prg-1* catalytic mutant animals (*prg-1*^*ADH*^) (*P*-values = 0.97 and 0.56, respectively), whereas in *prg-1(n4357)* loss of function mutants both *his-12* and *his-10* were strongly silenced (*P* < 0.0005) (Figure 5I). Thus, it is unlikely that PRG-1 is directly involved in histone 3′ end maturation. It is possible that PRG-1 recruits other factors to promote histone maturation. However, we did not observe extended 3′ ends on histone mRNAs in our RNA-seq data, arguing against this possibility (Figure 5A). Nonetheless, histones were among the most highly represented genes in *in vivo* PRG-1-mRNA crosslinking experiments (median gene rank: 371 out of 20 204 genes; rank range: 6–7391), pointing to a direct interaction between the piRNA machinery and histone mRNAs (Supplementary Table S9) (24,62).

*prg-1* mutants display a transgenerational loss of fertility (27). Because we analyzed *his-12* and *his-10* mRNA levels in the new CRISPR-Cas9 deletion strains used in this study directly after generating them, our results indicate that histone silencing occurs immediately upon loss of *prg-1*. It is possible, however, that the silencing becomes progressively stronger over multiple generations. To test this, we compared by qRT-PCR *his-12* and *his-10* expression in our fresh deletion allele of *prg-1* as soon as it was possible to obtain a homozygous line and then again after 10 generations of growth on a continuous supply of food at 20°C. There was no detectable difference in either *his-12* or *his-10* expression between 1–10 generations (*P*-values = 0.89 and 0.99, respectively) (Figure 5J). Therefore, it is unlikely that histone silencing in *prg-1* mutants is progressive over multiple generations, although it is still possible that it contributes to the transgenerational sterility of *prg-1* mutants through cumulative effects of reduced histone activity on gene expression across generations.

### piRNA target site abundance is not correlated with mRNA silencing

**Figure 6.**
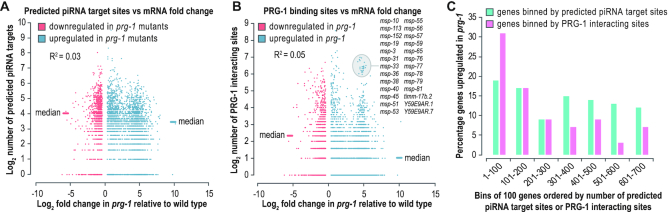
Correlation between piRNA target site abundance and mRNA silencing. (**A**) Scatter plot displaying each gene misexpressed in the distal gonads of *prg-1(n4357)* mutants as the log_2_ number of predicted piRNA target sites it contains (y-axis) versus its log_2_ fold-change in *prg-1* mutants (x-axis). (**B**) Scatter plot displaying each gene misexpressed in the distal gonads of *prg-1(n4357)* mutants as the log_2_ number of PRG-1 binding sites it contains (y-axis) versus its log_2_ fold-change in *prg-1* mutants (x-axis). (**C**) Bar plot displaying the percentage of genes upregulated in the distal gonads of *prg-1(n4357)* mutants in bins of genes ranked by either the number of predicted piRNA target sites or the number of PRG-1 interacting sites they contain. The top 700 genes in each category are in sequential bins of 100.

Two distinct approaches were recently used to identify piRNA targets in *C. elegans*. The first approach computed base-pairing rules for piRNA-target mRNA interactions to predict piRNA target sites genome-wide, and the second approach used *in vivo* crosslinking of PRG-1-piRNA complexes to target mRNAs to identify piRNA-mRNA interactions (23,24). To determine if mRNA upregulation in *prg-1* was correlated with potential targeting by piRNAs, we identified the number of predicted piRNA target sites and the number of PRG-1 binding sites on genes upregulated or downregulated in the distal gonads of *prg-1* mutants (Supplementary Tables S8 and S9). We did not observe a general correlation between mRNA fold-change in *prg-1* mutants and the number of predicted piRNA target sites or PRG-1 binding sites (*R*^2^ = 0.03 and 0.05, respectively) (Figure 6A and B). The median number of predicted target sites and PRG-1 binding sites was actually somewhat higher for genes downregulated in *prg-1* mutants than it was for genes upregulated (Figure 6A and B). This is consistent with previous work suggesting that neither method alone is predictive of piRNA-mediated gene silencing (62).

It is possible that many of the genes we identified as being upregulated are indirect targets, which could contribute to the lack of correlation between piRNA target sites and differential expression in *prg-1* mutants. We thus took an alternative approach in which we binned the top 700 genes with the highest numbers of predicted piRNA target sites or PRG-1 binding sites in increments of 100 genes and calculated the percentage in each bin that were upregulated in *prg-1* mutants. Based on the number of predicted piRNA target sites, there was only a modest difference in the percentage of genes that were upregulated in *prg-1* mutants across the seven bins, with 17% upregulated in the bin containing the top 100 genes and 12% upregulated in the bin containing the top 601–700 genes (Figure 6C). Of the top 100 genes ranked by PRG-1 interacting sites, 31% were upregulated in *prg-1* mutants and in subsequent bins the proportion trended downward, such that only 7% of genes in the bin containing the top 601–700 were upregulated in *prg-1* mutants (Figure 6C). This suggests that piRNA target site abundance and PRG-1 interacting sites have limited reliability in predicting piRNA-mediated gene silencing. We then examined a cluster of 26 genes that were both highly upregulated in *prg-1* mutants and contained a high number of PRG-1 interacting sites. Nearly all the genes within this cluster belong to a largely paralogous family of sperm proteins (Major Sperm Protein family), relating to our earlier observation that spermatogenic genes are upregulated in *prg-1* mutants and suggesting that at least some are directly regulated by piRNAs (Figure 6B).

### Correlation between 22G-RNA production and mRNA silencing

**Figure 7.**
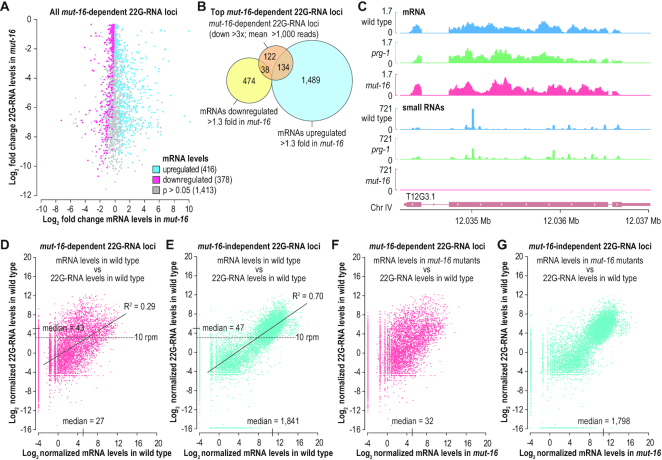
Relationship between 22G-RNAs and target mRNA expression. (**A**) Scatter plot displaying each *mut-16-*dependent 22G-RNA locus as a function of its log_2_ fold-change in 22G-RNA (y-axis) and mRNA (x-axis) levels in *mut-16(pk710)* mutants relative to wild type distal gonads. (**B**) Overlap between *mut-16*-dependent 22G-RNA loci producing >1000 normalized reads and mRNAs downregulated or upregulated >1.3× in *mut-16(pk710)* mutants relative to wild type distal gonads. (**C**) mRNA and small RNA read distribution across a representative *mut-16*-dependent 22G-RNA locus, T12G3.1, for which mRNA levels are unchanged in *mut-16(pk710)* mutants. For simplicity, strandedness is not shown. (**D, E**) Scatter plots displaying each annotated coding gene as a function of its log_2_ normalized 22G-RNA reads, categorized as *mut-16*-dependent (D) or *mut-16*-independent (E), in wild type animals (y-axes) versus mRNA reads in the distal gonads of wild type animals (x-axes). Median mRNA reads for genes that produce >10 normalized 22G-RNA reads (reads per million total mapped reads, rpm) are indicated on the x-axes. Median 22G-RNA reads are indicated on the y-axes. (**F, G**) Scatter plots displaying each annotated coding gene as a function of its log_2_ normalized 22G-RNA reads, categorized as *mut-16*-dependent (F) or *mut-16*-independent (G), in wild type animals (y-axes) versus mRNA reads in *mut-16(pk710)* mutant animals (x-axes). Median mRNA reads for genes that produce >10 normalized 22G-RNA reads (rpm) are indicated on the x-axes.

The relationship between WAGO-class 22G-RNAs and target mRNA expression is not well understood. To explore the role of 22G-RNAs in regulating gene expression in the germline, we compared small RNA and mRNA expression from *mut-16*-dependent 22G-RNA loci in wild type and *mut-16* mutants. Of the 2738 annotated gene loci depleted of 22G-RNAs by >1.3-fold in *mut-16* mutants, ∼81% were represented at sufficient levels for statistical analysis in our mRNA sequencing libraries from distal gonads. Of these, ∼19% were upregulated and ∼17% were downregulated in *mut-16* mutants (*P* < 0.05, no fold-change cutoff applied) (Figure 7A). For the remaining ∼64%, we did not detect a difference in mRNA levels in *mut-16* mutants (Figure 7A).

The modest and bidirectional effect we observed on *mut-16-*dependent 22G-RNA target mRNAs could reflect low-level, inconsequential small RNA production from the majority of WAGO-class 22G-RNA targets. Therefore, we focused on the *mut-16*-dependent loci with the highest abundance of 22G-RNAs: the 294 loci that produced >1,000 normalized 22G-RNA reads on average in our wild type distal gonad libraries and that were depleted >3-fold in *mut-16* mutant libraries. Surprisingly, only ∼46% of the mRNAs corresponding to the 294 22G-RNA loci were upregulated >1.3-fold in *mut-16* mutants, and ∼13% were instead downregulated >1.3-fold (Figure 7B). The remaining ∼41% were unaffected in *mut-16* mutants, despite loss of abundant 22G-RNAs (Figure 7B). The predicted coding gene T12G3.1, for example, produced very high levels of *mut-16*-dependent 22G-RNAs but its overall mRNA levels were not detectably changed in *mut-16* mutants (Figure 7C). These results indicate that WAGO-class 22G-RNA abundance is not a reliable indicator of RNA silencing.

Finally, we examined more generally the relationship between siRNA production and mRNA expression in the distal germline, including both *mut-16*-dependent and *mut-16*-independent 22G-RNA loci. Of the 6121 genes that yielded >10 normalized 22G-RNA reads (10 reads per million total mapped reads, rpm) in wild type animals, ∼28% were depleted of 22G-RNAs by >2-fold in *mut-16* mutants and are thus presumed to be WAGO targets (Supplementary Table S17). We observed a clear distinction in expression levels between mRNAs that produced 22G-RNAs depleted >2-fold in *mut-16* mutants and those that did not (Figure 7D and E). From the presumptive WAGO targets that yielded >10 normalized reads (rpm), the median normalized mRNA read counts was only 27 (∼4.75 on a log_2_ scale) (Figure 7D). In contrast, the median mRNA reads for *mut-16*-independent 22G-RNA loci that yielded >10 normalized small RNA reads was 1,841, despite nearly identical median levels of 22G-RNA reads from *mut-16*-dependent and *mut-16*-independent loci (∼43 versus ∼47) (Figure 7D and E). These *mut-16*-independent 22G-RNA loci are presumably CSR-1 targets as this is the only other characterized class of 22G-RNAs. Consistent with the weak correlation between *mut-16*-dependent 22G-RNA production and mRNA silencing noted above, the median reads for *mut-16*-dependent 22G-RNA target mRNAs was increased by only ∼19% in the distal gonads of *mut-16* mutants relative to wild type (Figure 7D and F). As predicted, the expression of genes yielding *mut-16*-independent 22G-RNAs, which are presumed to be CSR-1-class 22G-RNAs, was essentially unchanged in *mut-16* mutants (Figure 7E and G). Lastly, we observed a strong positive correlation between 22G-RNA levels and corresponding mRNA levels for *mut-16*-independent loci (*R*^2^ = 0.70) but to a much lesser extent for *mut-16*-dependent loci (*R*^2^ = 0.29) (Figure 7D and E). This supports the proposed role for the *mut-16*-independent branch of the 22G-RNA pathway involving CSR-1 in promoting germline gene expression (16–18). From these results, we conclude that WAGO-class 22G-RNAs are typically derived from poorly expressed genes and have little impact on the expression of most target mRNAs.

## DISCUSSION

### Regulation of gametogenesis by piRNAs and WAGO-class 22G-RNAs

Through a genome-wide parallel analysis of mRNA and small RNA defects in the distal gonads of *prg-1* and *mut-16* mutants, we uncovered wide-ranging roles for piRNAs and WAGO-class 22G-RNAs in shaping the transcriptome of the *C. elegans* distal germline. Widespread misexpression of gametic genes in *prg-1* and *mut-16* mutants points to a role for both classes of small RNA in controlling germ cell fate. The extent to which this is a direct effect and is not caused by other developmental defects is not clear. Regardless, it may help to explain the reduced fertility of *prg-1* and *mut-16* mutants (4–6,20). *prg-1* was previously implicated in regulating spermatogenesis, and the fertility defects of *prg-1* mutants are partially rescued by providing wild type sperm to *prg-1* mutant hermaphrodites (4). However, in L4 stage larvae, the stage at which wild type hermaphrodites are normally undergoing spermatogenesis, spermatogenic genes are downregulated (4). Nonetheless, our results demonstrating that sperm-enriched transcripts are upregulated in the distal gonads of adult *prg-1* mutants are consistent with previous results observed in tiling array experiments involving whole adult animals (5). Upregulation of spermatogenic genes in the distal gonad, which lacks gametes and should be fully transitioned to oogenesis, points to incomplete shutoff of sperm transcripts during oogenesis in *prg-1* mutants. Given that spermatogenic genes are also upregulated in *mut-16* mutants, albeit to a lesser extent, the role of *prg-1* in regulating spermatogenesis is likely linked to its function in routing mRNA targets into the WAGO-class 22G-RNA pathway (10,13).

### Roles of piRNAs and WAGO-class 22G-RNAs in regulating transposons

The reduced fertility in *prg-1* and *mut-16* mutants could also be caused by elevated levels of transposon mRNAs and a subsequent increase in mutagenic transposition events. Our data supports a prevalent role for *mut-16* and WAGO-class 22G-RNAs in silencing transposons, but a far more limited role for piRNAs. There was a very modest effect on transposon mRNA levels in *prg-1* mutants and 22G-RNA levels for most transposons were upregulated, rather than downregulated as would be predicted if piRNAs had a role in directing mRNAs into the WAGO-class 22G-RNA pathway. Nonetheless, consistent with previous studies, Tc3 mRNA levels were modestly upregulated in *prg-1* mutants and it was previously shown that Tc3 transposition rates are substantially higher in *prg-1* mutants (6). MIRAGE transposon mRNA levels were marginally affected in our datasets but were substantially upregulated in another study involving the same allele of *prg-1* (26). Some transposons identified previously as being upregulated in *prg-1* using qRT-PCR were also not affected in our datasets (10). These results can be reconciled in a model in which transposon desilencing in *prg-1* mutants is somewhat stochastic, possibly resulting from inconstant inheritance of the WAGO-class 22G-RNAs that provide a transgenerational memory of piRNA activity (11,12,14). Rearing conditions and many rounds of propagation could exacerbate the effect.

### Histone silencing in *prg-1* mutants

We observed a striking reduction in the levels of most histone mRNAs in *prg-1* mutants, which coincided with misrouting of histone mRNAs into the HRDE-1 nuclear RNAi pathway. Canonical replication-dependent histone mRNAs are distinct from most protein-coding mRNAs in that they are not thought to contain poly(A) signal sequences and poly(A) tails but rather contain a hairpin in their 3′UTRs that promotes cleavage and maturation of the 3′ end (60). In *C. elegans*, the U7 snRNA implicated in cleaving histones in other metazoans is absent, and it is not known how histone 3′ end maturation occurs (60). It is possible that PRG-1 promotes 3′ end cleavage and in its absence histone mRNAs are recognized as aberrant and thus routed into the HRDE-1 pathway. However, *prg-1* is clearly not essential for histone 3′ end formation, as many replication-dependent histone mRNAs were unaffected in *prg-1* mutants. Furthermore, we did not observe a difference in histone mRNA 3′ ends in our wild type and *prg-1* mutant sequencing datasets. The slicer activity of PRG-1 was also presumably not required for proper histone expression, which argues against a direct role in processing. Nonetheless, it is possible that other factors are redundant with *prg-1* in histone processing. Interestingly, histone mRNAs are also downregulated in *csr-1* mutants. CSR-1 appears to have a direct but unclear role in histone maturation (63). Perhaps CSR-1 and PRG-1 function redundantly to process histone mRNAs, which would be rather unusual given their seemingly opposite roles in regulating gene expression otherwise. Other Argonautes, such as the WAGOs, may also be involved in regulating histones, which could explain why we observed a modest reduction in some histone levels in *mut-16* mutants.

Transcription of the core histones is coupled to the cell cycle and therefore it is possible that defects in germ cell proliferation in the germlines of *prg-1* mutants is responsible for reduced histone mRNA levels (60). While this is certainly plausible, it does not explain why histone mRNAs are misrouted into the HRDE-1 RNAi pathway in *prg-1* mutants, nor does it explain why histone mRNAs are directly targeted by PRG-1, as suggested by *in vivo* crosslinking experiments (24). Consequently, the role of PRG-1 in regulating histones is likely at least partially direct and may impact proliferation of germline stem cells, which could explain the diminutive germlines of *prg-1* mutants.

### Relationship between 22G-RNAs and mRNA expression

The role of 22G-RNAs in regulating gene expression in *C. elegans* is not well understood. Nearly all distal germline-expressed genes produce 22G-RNAs (Figure 7D and E) (16,64), yet there are two distinct classes of 22G-RNAs that seem to act in opposition to one another. *mut-16*-dependent WAGO-class 22G-RNAs are thought to silence gene expression, whereas *mut-16*-independent CSR-1-class 22G-RNAs are thought to promote gene expression (22). Our data demonstrates that high levels of *mut-16*-independent 22G-RNAs is directly correlated with high-level gene expression, supporting a role for the CSR-1 branch of the 22G-RNA pathway in licensing genes for expression, (17,18). In contrast, the relationship between *mut-16*-dependent 22G-RNA production and gene expression is relatively weakly correlated and the majority of WAGO targets are poorly expressed, even in *mut-16* mutants. It is possible that the WAGO pathway imparts epigenetic modifications at target loci that somehow persist over multiple generations in the absence of 22G-RNAs. Alternatively, the WAGO pathway may selectively target poorly expressed genes as a means of combatting leaky transcription in the germline. Whatever the reason, these results point to a complex relationship between siRNA and mRNA expression and demonstrate that WAGO-class 22G-RNA production is not necessarily a good indicator of RNA silencing. A recent study exploring small RNA production in the *C. elegans* gonad concluded that 22G-RNA levels were inversely correlated with mRNA expression, which is not consistent with our results (64). The reason for this discrepancy may be that the authors relied on external mRNA sequencing datasets to complement their small RNA sequencing data, whereas our small RNA and mRNA data were generated from the same RNA samples.

### Additional roles for piRNAs and WAGO-class 22G-RNAs in gene regulation

We identified hundreds of genes misregulated in *prg-1* and *mut-16* mutants that did not fall into any of the specific categories we explored. For example, several genes involved in RNA silencing pathways were misexpressed in *prg-1* and *mut-16* mutants. The piRNA trimmer *parn-1*, for instance, was upregulated in *prg-1* and *mut-16* mutants, and the RNA helicase *eri-6/7*, required for ERGO-1-class 26G-RNA production, was strongly downregulated in *mut-16* mutants (65,66). It will be important to investigate the roles of piRNAs and WAGO-class 22G-RNAs in regulating specific genes identified in this study as being misregulated in *prg-1* and *mut-16* mutants (see Supplementary Tables S8, S9, S12, and S13 for comprehensive lists of misregulated genes; see https://www.montgomerylab.org/resources.html to visualize the data in a genome browser).

Additional roles for piRNAs and WAGO-class 22G-RNAs in regulating gene expression in the germline will likely emerge from analysis of animals grown under non-optimal conditions. At 25°C, for example, the fertility defects of *prg-1* and *mut-16* mutants are exacerbated. Our characterization of transposon silencing in whole animals did not reveal any substantial differences in transposon silencing in *prg-1* or *mut-16* mutants grown at 25°C compared to animals grown at 20°C. However, we limited our analysis to transposons as *prg-1* and *mut-16* mutants grown at 25°C have developmental defects that could confound differential expression results and in particular increase the likelihood of false positives caused by indirect effects on gene expression.

This study provides a valuable framework for exploring the roles of small RNAs in regulating gene expression as it relates to development, genome defense, and epigenetic inheritance in *C. elegans*. The results will likely help to uncover shared and conserved roles for small RNAs in other animals as well.

## DATA AVAILABILITY

All raw high-throughput sequencing data and counts tables described here have been deposited to the Gene Expression Omnibus (GEO) and is available under accession number GSE141243.

## Supplementary Material

gkz1178_Supplemental_Files
