# Supplementary material for: Widespread roles for piRNAs and WAGO-class siRNAs in shaping the germline transcriptome of Caenorhabditis elegans
Source: Nucleic Acids Res. 2019 Dec 24;48(4):1811–27. doi: 10.1093/nar/gkz1178 (PMC7038979; doi:10.1093/nar/gkz1178)
Supplement: gkz1178_Supplemental_Files [file gkz1178_supplemental_files.zip › ReedSupplementalFinal.pdf]

Nucleic Acids Research

Supplementary Data

Widespread roles for piRNAs and WAGO-class siRNAs in shaping the germline transcriptome of *Caenorhabditis elegans*

Kailee J. Reed[1,2], Joshua M. Svendsen[1,2], Kristen C. Brown[1,2], Brooke E. Montgomery[1], Taylor N. Marks[1], Tarah Vijayasarathy[1], Dylan M. Parker[3], Erin Osborne Nishimura[3], Dustin L. Updike[4], and Taiowa A. Montgomery[1*]

[1]Department of Biology, Colorado State University, Fort Collins, CO 80523, USA

[2]Cell and Molecular Biology Program, Colorado State University, Fort Collins, CO 80523, USA

[3]Department of Biochemistry and Molecular Biology, Colorado State University, Fort Collins, CO 80523, USA

[4]Mount Desert Island Biological Laboratory, Bar Harbor, ME 04672, USA

*Correspondence: tai.montgomery@colostate.edu

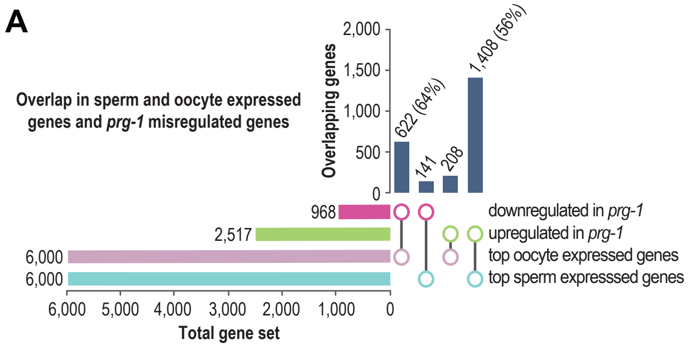

Figure S1. Misregulated gene sets in *prg-1* and *mut-16* are enriched for oocyte and sperm expressed genes. **(A)** UpSet plots display the overlap in genes upregulated or downregulated in *prg-1(n4357)* and the top 6,000 most highly expressed in sperm or oocytes. The percentages shown are for the gene sets downregulated or upregulated in *prg-1(n4357)*. (**B)** UpSet plots display the overlap in genes upregulated or downregulated in *mut-16(pk710)* and the top 6,000 most highly expressed in sperm or oocytes. The percentages shown are for the gene sets downregulated or upregulated in *mut-16(pk710)*.

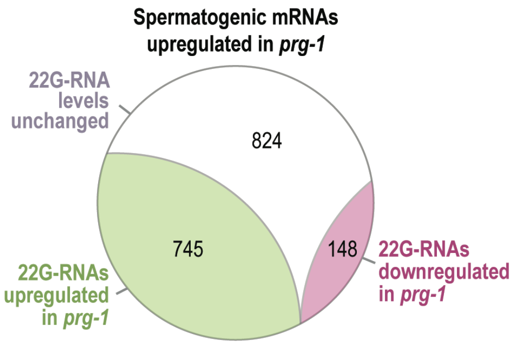

Figure S2. Changes in 22G-RNA levels from spermatogenic mRNAs upregulated in *prg-1* or *mut-16* mutants. Partial Venn diagrams display spermatogenic genes as the overlap in upregulated mRNAs in either *prg-1(n4357)* or *mut-16(pk710)* and upregulated and downregulated 22G-RNAs.

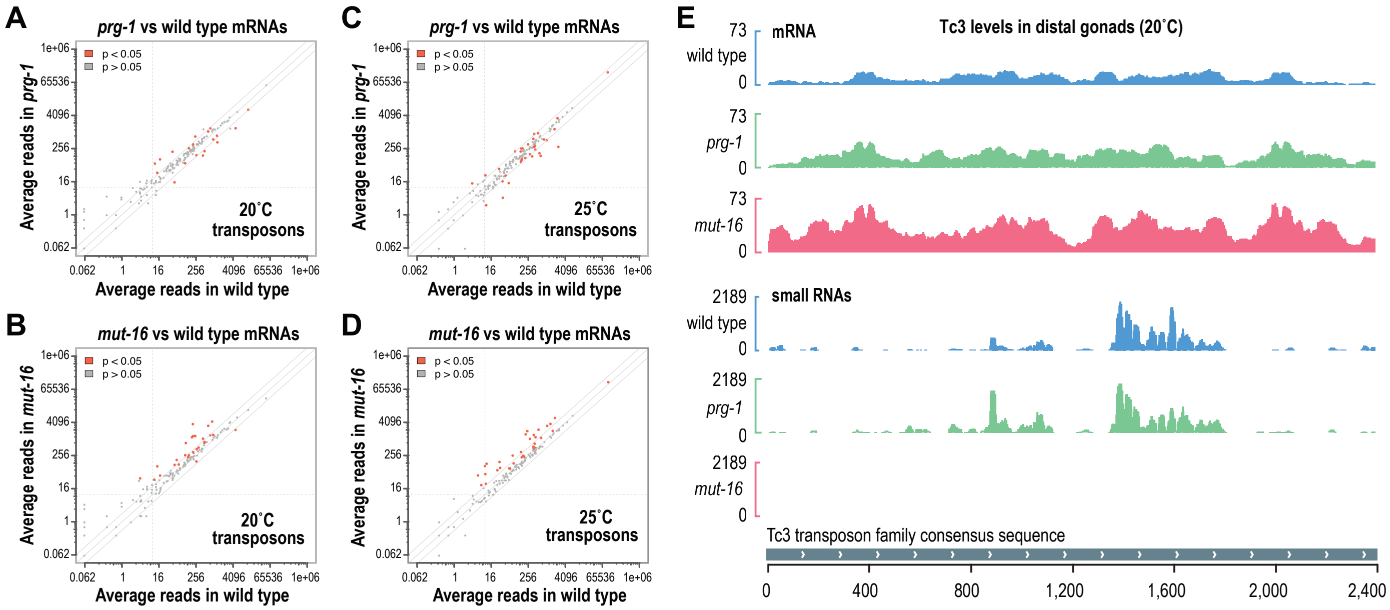

Figure S3. Transposon misexpression in *prg-1* and *mut-16* mutant whole animals. **(A)** Each transposon family (152 total) is plotted as a function of mRNA reads in *prg-1(n4357)* (y-axis) vs wild type (x-axis) from whole animals grown at 20˚C. **(B)** Each transposon family (152 total) is plotted as a function of mRNA reads in *prg-1(n4357)* (y-axis) vs wild type (x-axis) from whole animals grown at 25˚C. **(C)** Each transposon family (152 total) is plotted as a function of mRNA reads in *mut-16(pk710)* (y-axis) vs wild type (x-axis) from whole animals grown at 20˚C. **(D)** Each transposon family (152 total) is plotted as a function of mRNA reads in *mut-16(pk710)* (y-axis) vs wild type (x-axis) from whole animals grown at 25˚C. **(E)** mRNA and small RNA read distribution across the Tc3 transposon consensus sequence in wild type animals and *prg-1(n4357)* and *mut-16(pk710)* mutants. Data is from distal gonads dissected from animals grown at 20˚C.

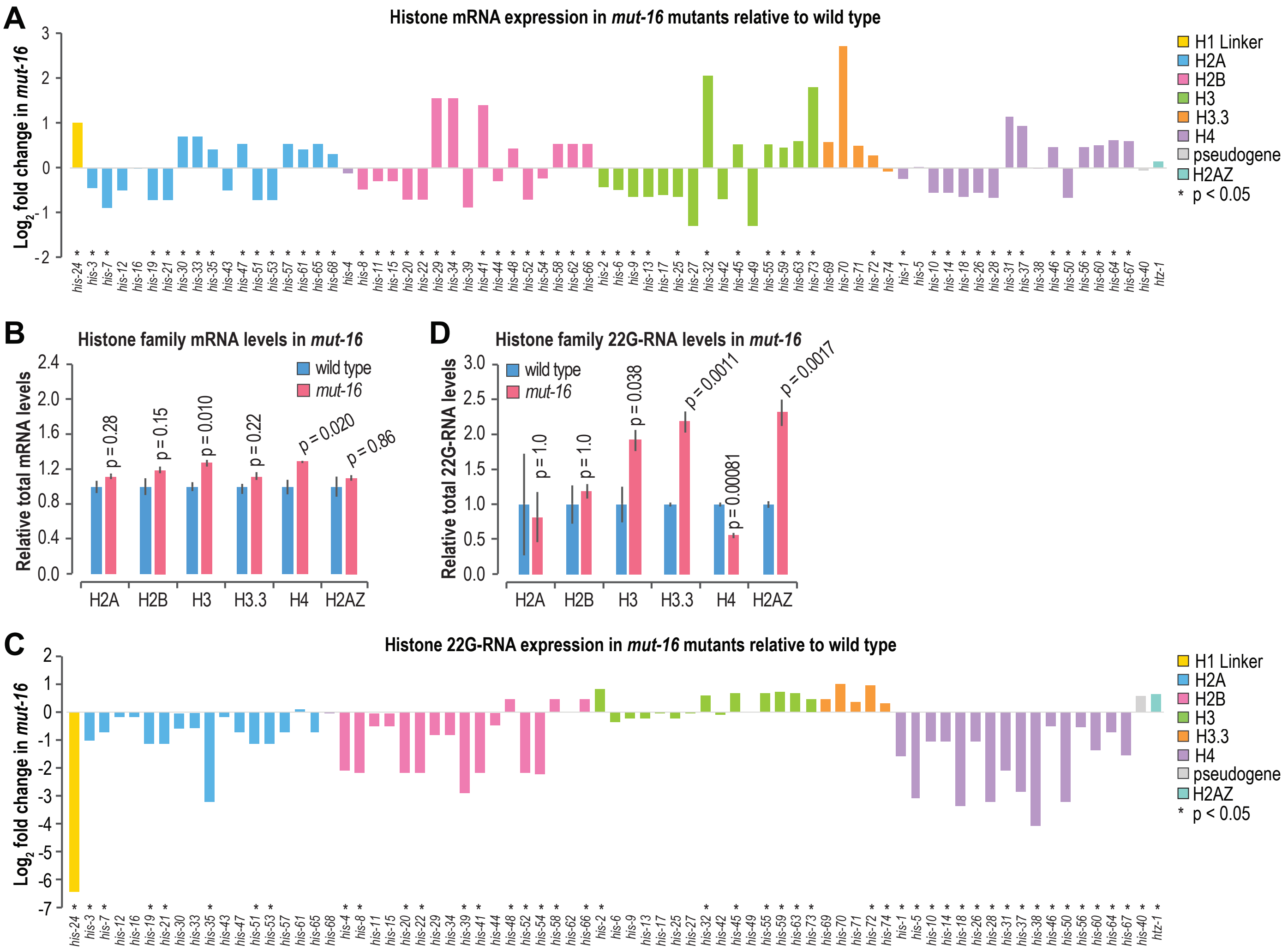

Figure S4. Histone misexpression in *mut-16* mutants. **(A)** Bar plot displaying each histone gene as a function of its $\log_2$-fold change in mRNA expression in *mut-16(pk710)* mutants relative to wild type. Bars are colored by histone family as indicated in the key. **(B)** Total histone family mRNA levels in *mut-16(pk710)* mutants relative to wild type animals. Error bars show standard deviation (n = 3 biological replicates). **(C)** Bar plot displaying each histone gene as a function of its $\log_2$-fold change in 22G-RNA expression in *mut-16(pk710)* mutants relative to wild type. Bars are colored by histone family as indicated in the key. **(D)** Total histone family 22G-RNA levels in *mut-16(pk710)* mutants relative to wild type animals. Error bars show standard deviation (n = 3 biological replicates).
